# Co_3_O_4_ Nanostructured Sensor for Electrochemical Detection of H_2_O_2_ as a Stress Biomarker in Barley: Fe_3_O_4_ Nanoparticles-Mediated Enhancement of Salt Stress Tolerance

**DOI:** 10.3390/mi15030311

**Published:** 2024-02-24

**Authors:** Vjaceslavs Gerbreders, Marina Krasovska, Eriks Sledevskis, Irena Mihailova, Valdis Mizers

**Affiliations:** G. Liberts’ Innovative Microscopy Centre, Department of Technology, Institute of Life Sciences and Technology, Daugavpils University, Parades Street 1a, LV-5401 Daugavpils, Latvia; vjaceslavs.gerbreders@du.lv (V.G.); eriks.sledevskis@du.lv (E.S.); irena.mihailova@du.lv (I.M.); valdis.mizers@du.lv (V.M.)

**Keywords:** nanostructured electrochemical sensor, cobalt oxide nanopetals, hydrogen peroxide, salt stress, oxidative stress, stress tolerance, barley, iron oxide nanoparticles

## Abstract

This research investigates the enhancement of barley’s resistance to salt stress by integrating nanoparticles and employing a nanostructured Co_3_O_4_ sensor for the electrochemical detection of hydrogen peroxide (H_2_O_2_), a crucial indicator of oxidative stress. The novel sensor, featuring petal-shaped Co_3_O_4_ nanostructures, exhibits remarkable precision and sensitivity to H_2_O_2_ in buffer solution, showcasing notable efficacy in complex analytes like plant juice. The research establishes that the introduction of Fe_3_O_4_ nanoparticles significantly improves barley’s ability to withstand salt stress, leading to a reduction in detected H_2_O_2_ concentrations, alongside positive impacts on morphological parameters and photosynthesis rates. The developed sensor promises to provide real-time monitoring of barley stress responses, providing valuable information on increasing tolerance to crop stressors.

## 1. Introduction

The increasing global demand for food, coupled with the challenges posed by climate change, pests, and resource limitations, has spurred a growing interest among scientists in exploring innovative solutions to mitigate crop losses and enhance agricultural productivity [1,2,3,4]. Nanoparticles, with their unique properties at the nanoscale, have emerged as a promising avenue for addressing these challenges in agriculture [5,6,7]. Scientists are exploring the application of nanoparticles to improve nutrient delivery [8], enhance pest [9,10] and disease management [11,12], optimize water use efficiency [13], and bolster crop resilience to environmental stresses [14,15].

Among various environmental stressors, soil salinity emerges as a formidable challenge, adversely affecting plant growth and development, and ultimately reducing agricultural yield [16,17]. Salinity induces osmotic stress, leading to the dehydration of plant cells, wilting, and stunted growth [18,19]. It disrupts ion balance within plant tissues, impairing nutrient uptake and metabolic functions [20,21,22]. This imbalance hampers the uptake of crucial nutrients such as nitrogen, phosphorus, and potassium [23,24], while promoting the accumulation of reactive oxygen species (ROS), which cause oxidative stress and cellular damages [25,26,27,28]. Furthermore, salinity impairs photosynthesis, a critical process for energy production and biomass accumulation, thereby diminishing plant growth and yields [29,30]. However, it is noteworthy that certain plant varieties display varying degrees of salt tolerance, which has led researchers to develop and study salt-tolerant varieties to mitigate the impacts of salinity on crop productivity [31,32,33,34].

In this context, Fe_3_O_4_ nanoparticles (magnetite nanoparticles) have garnered attention for their potential to mitigate salt stress in plants [35,36,37,38]. These nanoparticles offer several mechanisms for alleviating the adverse effects of salinity, including the sequestration of sodium ions, which are primarily responsible for salt stress [39,40]. Furthermore, Fe_3_O_4_ nanoparticles can enhance nutrient uptake and improve water availability in saline soils, thus supporting plant growth under stress conditions [41,42,43]. Additionally, they may bolster plant defenses against oxidative stress by stimulating the production of antioxidants and stress-response molecules [44,45], and positively affect soil microbial communities, essential for nutrient cycling and soil health [46].

Hydrogen peroxide (H_2_O_2_) detection emerges as a critical method for assessing plant oxidative stress levels, employing various analytical techniques, each with its unique advantages and limitations. While colorimetric and fluorometric assays offer simplicity and sensitivity, they suffer from potential interference in complex matrices [47,48]. Titration methods provide reliable results but are time-consuming and less sensitive for trace analyses [49]. Enzymatic assays, though specific, require complex protocols [50,51]. In contrast, electrochemical sensors present a promising alternative, offering high sensitivity, selectivity, real-time monitoring capabilities, and the potential for miniaturization, making them suitable for diverse applications, including environmental monitoring and point-of-care diagnostics [52,53,54]. Various materials are utilized in electrochemical sensor development for H_2_O_2_ detection, each offering unique attributes. These include metal oxides like titanium dioxide (TiO_2_) [55,56], zinc oxide (ZnO) [57,58,59], and indium oxide (In_2_O_3_) [60], as well as carbon-based materials such as carbon nanotubes [61,62] and graphene [63]. Noble metals like gold (Au) [64] and platinum (Pt) [65], along with metal nanoparticles like silver (Ag) [66], palladium (Pd) [67], and copper (Cu) [68], are also employed. Polymer nanocomposites incorporating nanoparticles or nanotubes contribute to material diversity [69]. However, Co_3_O_4_ emerges as an advantageous choice due to its exceptional catalytic activity, ensuring enhanced electrochemical reactions critical for H_2_O_2_ detection. Its chemical and electrochemical stability ensures sensor reliability and longevity. Moreover, the cost-effectiveness of Co_3_O_4_ and its abundance make it practical for large-scale sensor production [70]. Additionally, high surface area, achievable through nanostructuring, facilitates increased interaction with analytes, resulting in heightened sensitivity and responsiveness in H_2_O_2_ detection. Its versatility in various nanostructured morphologies allows tailored customization to meet specific sensor requirements [71,72,73]. This study focuses on creating an electrochemical sensor utilizing petal-shaped nanostructures of Co_3_O_4_ for the detection of H_2_O_2_ released in barley subjected to salt stress. Additionally, the research aims to investigate the development of salt stress tolerance in barley samples, leading to a potential reduction in the released H_2_O_2_ concentration. The study also explores the impact of Fe_3_O_4_ nanoparticles on this process, aiming to discern their influence on salt stress tolerance and subsequent H_2_O_2_ levels in barley samples.

## 2. Materials and Methods

### 2.1. Materials

Iron(III) chloride hexahydrate (FeCl_3_·6H_2_O, CAS number: 10025-77-1), Iron(II) chloride tetrahydrate (FeCl_2_·4H_2_O, CAS number: 13478-10-9), Ammonium hydroxide solution (NH_4_OH, 32%, CAS number: 1336-21-6), Cobalt(II) nitrate hexahydrate (Co(NO_3_)_2_·6H_2_O, CAS number: 10026-22-9), urea (NH_2_CONH_2_, CAS number: 57-13-6), sodium chloride (NaCl, CAS number: 7647-14-5), potassium nitrate (KNO_3_, CAS number: 7757-79-1), glucose (C_6_H_12_O_6_, CAS number: 50-99-7) citric acid (HOC(COOH)(CH_2_COOH)_2_, CAS number: 77-92-9), ascorbic acid (C_6_H_8_O_6_, CAS number: 50-81-7), and hydrogen peroxide solution (H_2_O_2_, 30%, CAS number: 7722-84-1) were procured from Merck. All reagents demonstrated a purity level of at least 99.8%. Iron wires with a thickness of 2 mm (99.9% purity) were sourced from Sigma-Aldrich (St. Louis, MO, USA). Ag/AgCl wire was acquired from A-M Systems, Sequim, WA, USA. Carbon rods (5 mm diameter) were obtained from Sigma-Aldrich. Barley seeds (*Hordeum vulgare* L. “Marthe”) were acquired from an Institute of Agricultural Resources and Economics, Stende Research Center (Priekuli, Latvia). A universal peat substrate for seedlings cultivation (Durpeta, LT, Šepeta, Lithuania) was purchased at a local store. Distilled water used in the experiments was produced in the laboratory.

### 2.2. Synthesis and Characterization of Fe_3_O_4_ Nanoparticles

The nanoparticles were synthesized using the co-precipitation (Massart) method described by us in the previous publication [74]. This method makes it possible to obtain small nanoparticles suitable for plants processing. In this process, 0.2334 g of FeCl_3_·6H_2_O and 0.0858 g of FeCl_2_·4H_2_O were used for 100 mL of distilled water. Subsequently, 0.54 mL of 25% NH_4_OH was added dropwise to the solution using a pipette under continuous manual stirring. As a result of this reaction, 72 mg of a black precipitate is obtained. The resulting nanostructures were stabilized using an aqueous solution of citric acid (40 mg·mL^−1^, 2 mL). The resulting Fe_3_O_4_ precipitate was separated from solution with a permanent magnet and washed several times with distilled water to eliminate residual chemicals until the solution becomes transparent. The total hydrothermal synthesis process of Fe_3_O_4_ nanoparticles can be represented by Equation (1):

Fe^2+^ + 2Fe^3+^ + 8OH^−^ = Fe_3_O_4_↓ + 4H_2_O (1)

The morphology and size of the Fe_3_O_4_ nanoparticles was analyzed using Field Emission Scanning Electron Microscopy (FESEM) (MAIA 3, Tescan, Brno, Czech Republic) and Atomic Force Microscope (AFM) (NX 10, Park Systems Corp., Suwon, Republic of Korea). The chemical composition was investigated using an EDS installation (Inca, Oxford Instruments, Oxford, UK). The SEM image reveals that the Fe_3_O_4_ powder is composed of agglomerates of individual nanoparticles. AFM images show that the resulting nanoparticles have a spherical shape and an average nanoparticle size of 10 nm. The results of these studies are presented in our previous publication [75].

### 2.3. Barley Seedling Cultivation and Sample Preparation

A universal peat-containing substrate was employed for seed germination and growth. During the initial week of seed germination and the early stages of seedling development, all containers received daily watering with 20 mL of deionized water. Starting from the second week, to investigate the effects of salt stress and the development of salt stress tolerance under the influence of Fe_3_O_4_ nanoparticles, the samples were divided into five groups, each comprising four containers. The first group served as the control and received daily irrigation with 20 mL of deionized water per container. The second group was subjected to salt stress and, instead of deionized water, received a daily irrigation of 20 mL per container with a 0.2 M aqueous solution of NaCl. The third group received a daily 20 mL per container of an aqueous solution of Fe_3_O_4_ nanoparticles at a concentration of 72 mg·L^−1^. The fourth group was irrigated with a 0.2 M NaCl solution to which Fe_3_O_4_ nanoparticles were added, maintaining the initial concentration of 72 mg·L^−1^. The fifth group was also irrigated with a 0.2 M solution of NaCl and nanoparticles, but the nanoparticle concentration was reduced by half, amounting to 36 mg·L^−1^. This irrigation regimen was sustained for an additional three weeks. Other growth parameters, such as temperature (22 °C), humidity (50%), and illumination, were maintained the same for all samples.

At the end of the one-month period, morphological distinctions among the barley samples were evaluated via control measurements. Measurements included the length of the first leaf determination, the total length of the seedling, and the total green weight and dry weight per ten random plants from the studied group of samples. Harvesting of barley samples for chlorophyll and H_2_O_2_ measurements occurred two times in the third and fourth weeks of growth. The leaves were cut into 3–5 mm pieces, crushed using a mortar and pestle to break down hard plant tissues and facilitate the extraction process, and then placed in a container with liquid for extracting. For optical measurements, 125 mg of green mass per 5 mL of 96% ethanol were used, while for electrochemical measurements, 10 g of green mass per 250 mL of 0.1 M NaOH were employed. The samples were placed in a cool, dark location overnight to facilitate extraction. The barley extracts were filtered through filter paper to remove solid discolored plant tissues and then the barley extracts were used for measurements. In addition, a portion of the plants from each study group was dried in an oven and ground into powder using a mortar and pestle to perform EDS microanalysis in order to determine the content of trace elements in the samples.

### 2.4. Optical Mesurements

The quantification of chlorophyll serves as a vital parameter in assessing plant health, with its reduction being a key indicator of stress impact. In this investigation, the chlorophyll content of both photosystem II (PSII) and photosystem I (PSI) was examined by extracting chlorophyll from the leaves of untreated, salt stress-exposed and Fe_3_O_4_ nanoparticle-exposed barley seedlings.

To assess the effectiveness of Fe_3_O_4_ nanoparticles (nPs) in reducing the effect of salt stress on barley seedlings, measurements of the chlorophyll spectrum were taken from plants exposed to NaCl for two weeks and to NaCl for three weeks.

For the analysis, an extract, detailed in the previous section, was prepared and transferred into a 5 mL transparent cuvette for measurement. Each treatment group underwent five replicate measurements. A UV-Visible two-beam spectrophotometer, specifically the SHIMADZU UV-2550PC (Shimadzu Corporation, Kyoto, Japan), was employed for sample analysis. The determination of chlorophyll and carotenoid content utilized Arnon’s Equations (2)–(5) [39,76] by determining the absorbance values from the peaks on the obtained absorption graph.

Chlorophyll a (mg/g) = [12.7 × A663 − 2.69 × A645] × V/(1000 × W)(2)

Chlorophyll b (mg/g) = [22.9 × A645 − 4.68 × A663] × V/(1000 × W)(3)

Total chlorophyll (mg/g) = [20.2 × A645 + 8.02 × A663] × V/(1000 × W)(4)

Carotenoid (mg/g) = [A480 + 0.114 × A663 − 0.638 × A645] × V/(1000 × W)(5)

where Vis the volume of the extract in mL; W is the weight of fresh leaves in g; and A663, A645, A480 are solution absorbances at a specified wavelength.

### 2.5. Co_3_O_4_ Nanostructured Electrode Preparation and Electrochemical Measurements for H_2_O_2_ Detection

H_2_O_2_ belongs to ROS, so heightened concentrations signify the onset of oxidative stress in the plant. This underscores the importance of determining the concentration of this substance as a crucial measurement to assess the degree of the impact of stress factors on plant samples. For this purpose, we have developed a highly selective and sensitive electrochemical sensor based on Co_3_O_4_ nanostructures.

The process of synthesizing wire-like Co_3_O_4_ nanostructures from cobalt chloride precursor and developing an electrochemical sensor based on these nanostructures for detecting H_2_O_2_ in real samples is described in detail in our previous publications [77,78].

This publication established the high selectivity of this sensor for H_2_O_2_ determination, even in the presence of common interferents, making the sensor well-suited for analyzing real plant samples with complex chemical compositions. Specifically, the sensitivity of the Co_3_O_4_ electrode was determined to be 505.11 A·mM^−1^, and the calculated limit of detection (LOD) was found to be 1.05 μM.

Nonetheless, in the course of this publication, we encountered a challenge related to stabilizing the resulting electrode in solutions containing plant elements. This issue led to some inaccuracies when working with samples featuring low concentrations of the analyte. To address this concern, we undertook research focused on investigating the operational characteristics of a nanostructured Co_3_O_4_ sensor by altering the morphology of the nanostructured coating from fiber-like to petal-like. In pursuit of this objective, we substituted the chlorine-containing cobalt precursor with a nitrate-containing one during the synthesis process, while keeping the remainder of the synthesis process unchanged.

Electrodes coated with nanostructured Co_3_O_4_ were prepared through a two-step method including hydrothermal synthesis followed by thermal decomposition. Utilizing iron wire as the substrate for obtaining wire electrodes, the samples underwent a pre-synthesis treatment involving immersion in 0.1 M HCl, with the aim to enhance the electrode surface roughness, then were cut into 6 cm long segments and rinsed with water and ethanol. For the hydrothermal synthesis, an equimolar solution of 0.1 M Co(NO_3_)_2_·6H_2_O and NH_2_CONH_2_ was added to 80 mL of distilled water stirring constantly until the reagents are completely dissolved. The tempered borosilicate glass beaker, containing the growth solution and pretreated wire samples, was placed in a laboratory programmable oven and kept for 5 h at 95 °C, in order to obtain a nanostructured Co(OH)_2_ coating. Following the beaker cooling to room temperature, nanostructured electrodes underwent multiple washes with distilled water to eliminate residual reagents, followed by drying at 90 °C for 3 h and 1 h of annealing at 450 °C for the thermal decomposition of Co(OH)_2_, yielding Co_3_O_4_ nanostructures

The standard reactions associated with the formation of the nanoporous Co_3_O_4_ nanostructure can be described with Equations (6)–(10) [79]:

Co(NO_3_)_2_ → Co^2+^ + 2NO_3_^−^ (6)

Co (NH_2_)_2_ + 2H_2_O → 2NH_3_ + CO_2_↑ (7)

NH_3_ + H_2_O → NH_4_^+^ + OH^−^ (8)

Co^2+^ + 2OH^−^ → Co(OH)_2_ (9)

3Co(OH)_2_ → Co_3_O_4_ + 2H_2_O + H_2_↑ (10)

The H_2_O_2_ detection mechanism is based on the following principles. In the case of the nanostructured Co_3_O_4_ electrode, the nanostructures acted as a catalyst, facilitating the decomposition of H_2_O_2_ into CoOOH and H_2_O. The Co_3_O_4_ surface provided a large number of active sites where the oxidation process of H_2_O_2_ took place, causing the formation of CoOOH.

The catalytic reactivity was prominently observed upon the introduction of H_2_O_2_ into the test solution, causing a significant amplification in the amplitude of the peaks presented on the CV graphs and associated with oxidation and reduction processes. These two reversible reactions and detection mechanism can be described as follows [73,80,81]:

Co_3_O_4_ + OH^−^ + H_2_O → 3CoOOH + e^−^ (11)

CoOOH + OH^−^ → CoO_2_ + H_2_O + e^−^ (12)

**Figure 1:**
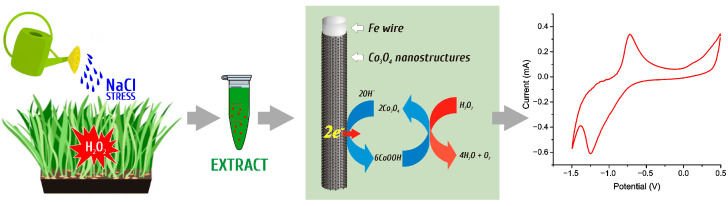
Schematic representation of the process of electrochemical determination of H_2_O_2_ in barley samples using a nanostructured Co_3_O_4_ electrode.

To study the operating features of the sensor, measurements were carried out according to the scheme described in detail in our previous publication [82]. The detection process is shown schematically in Figure 1.

Electrochemical measurements were carried out using a custom-made electrochemical cell, including an Fe/Co_3_O_4_ nanostructured working wire electrode, carbon counter electrode, and Ag/AgCl reference electrode and 0.1 M NaOH supporting electrolyte (pH = 13).

Cyclic voltammetry (CV) experiments were conducted with a voltage range spanning from −1.3 V to 0.5 V vs. Ag/AgCl, employing E_start_ = 0 V and a scan rate of 100 mV·s^−1^.

To evaluate electrode sensitivity, varied H_2_O_2_ concentrations ranging from 200 μM to 2 mM were introduced into the supporting electrolyte and CV graphs were obtained. The impact of scanning speed and the pH of the supporting electrolyte on the electrochemical response was also investigated.

In the current response analysis, a constant −1.2 V voltage was applied to the cell, and the resultant current was measured. These potential values corresponded to the peak positions on the CV graphs. Measurements were conducted in a 0.1 M NaOH supporting electrolyte, commencing without H_2_O_2_. After a stabilization period of 120 s, 25 μM portions of H_2_O_2_ were successively added every 120 s. To establish a calibration curve, H_2_O_2_ concentrations ranging from 25 μM to 7 mM were introduced. Stirring was maintained using a magnetic stirrer integrated in a water bath, allowing it to maintain a consistent temperature of 25 °C during all times of measurement.

Given that plant juice is a complex matrix comprising solid cellular structures, organic acids, sugars, and more, it is imperative to mitigate false increases in electrochemical response arising from interfering substances when developing an electrochemical sensor for such analytes. To address this, the electrode underwent interference testing by introducing into the supporting electrolyte 100 μM portions of NaCl, KNO_3_, glucose, citric acid, and ascorbic acid.

In the investigation of real samples, a 0.1 M NaOH-based barley extract was utilized and chronoamperograms were taken. Since the amount of H_2_O_2_ released in barley samples under stress factors is unknown, several known concentrations of H_2_O_2_ were added to the extract manually during measurements, and chronoamperograms were recorded. After requisite calculations based on the calibration graph obtained for the 0.1 M NaOH supporting electrolyte, the concentration of the identified H_2_O_2_ was determined. The amount of H_2_O_2_ released in plants was defined as the difference between the total amount of H_2_O_2_ found in solution and known amount of H_2_O_2_ that was artificially added performing the measurement process. For one measurement, 70 mL of analyte was used.

For data on the identified peroxide amount, averaged results from multiple sample batches were utilized.

## 3. Results and Discussion

**Figure 2:**
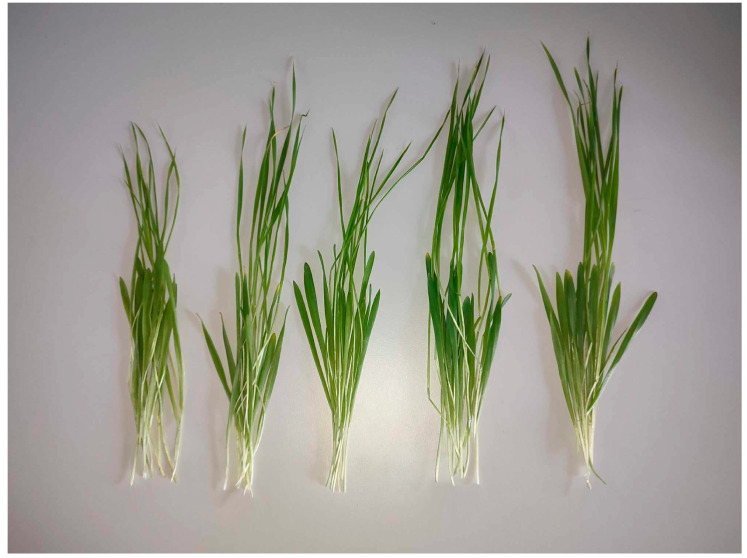
Barley samples, 10 seedlings from each group. From left to right: control sample, NaCl sample, nPs 100% sample, nPs 50%/NaCl sample, and nPs 100%/NaCl sample. The samples were grown for one week under water irrigation and for four weeks, of which one week was watered and three weeks were exposed to salt stress and Fe_3_O_4_ nanoparticles.

Figure 2 shows barley samples on the day of cutting (total growth time 4 weeks). The samples are arranged in the following sequence (from left to right): control, treated with 0.2 M NaCl (further mentioned as NaCl), treated with 72 mg·L^−1^ Fe_3_O_4_ nPs (further mentioned as nPs 100%), treated with 36 mg·L^−1^ Fe_3_O_4_ nPs and 0.2 M NaCl (further mentioned as nPs 50%/NaCl), and treated with 36 mg·L^−1^ Fe_3_O_4_ nPs and 0.2 M NaCl (further mentioned as nPs 100%/NaCl). The results of morphological measurements are summarized in Table 1.

Table 1 indicates that there is no significant difference in the length of the first leaf; however, the smallest value is observed in samples treated with NaCl, while the largest is seen in the sample treated with nPs 100%/NaCl. It is noteworthy that in samples concurrently treated with NaCl and nanoparticles, there is a notable increase in the total length of the green part (the combined length of the first and second leaves, measured from the node at the beginning of the first leaf to the tip of the second). This increase is approximately 2–4 cm more than in samples treated solely with NaCl.

Comparing the fresh weight of 10 plant samples, it is evident that NaCl-treated plants exhibit the lowest value. Nonetheless, when compared to the control sample and the sample treated only with nanoparticles, this difference is deemed insignificant. Notably, special attention should be given to samples treated simultaneously with NaCl and nanoparticles. Their fresh weight is approximately 0.6 g greater than other samples, and this effect persists even when the concentration of nanoparticles is halved. Furthermore, after drying, the dry weight is nearly identical for all samples.

**Figure 3:**
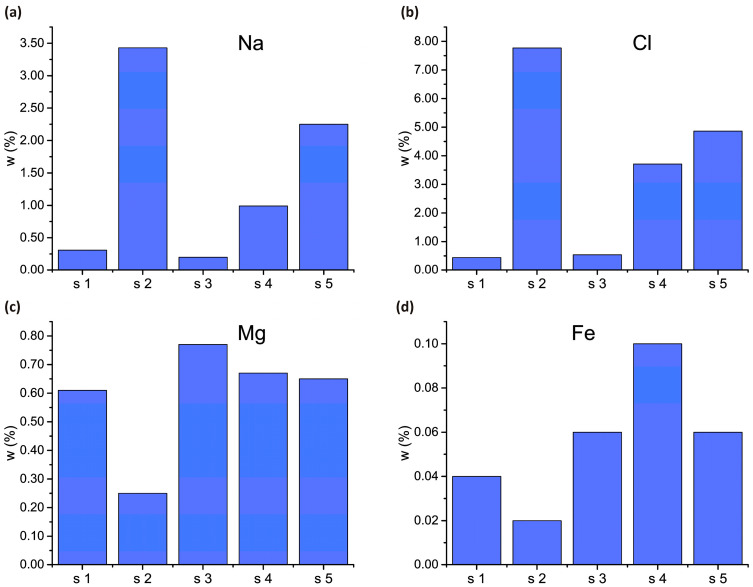
Content of microelements in barley samples (in weight percent). Here s1 is the control sample, s2 is the NaCl sample, s3 is the nPs 100% sample, s4 is the nPs 50%/NaCl sample, and s5 is the nPs 100%/NaCl sample. Data obtained from EDS microanalysis, where (**a**) Na content (**b**) Cl content (**c**) Mg content (**d**) Fe content The microanalysis results of the NaCl sample reveal that exposure to salt stress leads to a reduction in the concentration of several vital elements for plant functions, including Mg, C, P, Ca, and Fe, in comparison to the control sample. Of particular significance are Mg and Fe, given their crucial roles in the process of photosynthesis. Additionally, a notable surplus of Na and Cl was observed in the NaCl sample when compared to control samples. The introduction of Fe_3_O_4_ nanoparticles into the irrigation solution containing NaCl (samples 4 and 5) results in both an elevation of Mg, C, P, Ca, and Fe levels to a range comparable with the control sample and a reduction in the concentrations of Na and Cl. The obtained results clearly indicate that the incorporation of Fe_3_O_4_ nanoparticles positively influences the content of essential microelements under salt stress conditions. Simultaneously, it diminishes the concentrations of Na and Cl, signifying the development of tolerance to salt stress.

Figure 3 shows the microanalysis results for the most relevant elements for this experiment. The full results of the microanalysis are presented in Table A1 in Appendix A.

**Figure 4:**
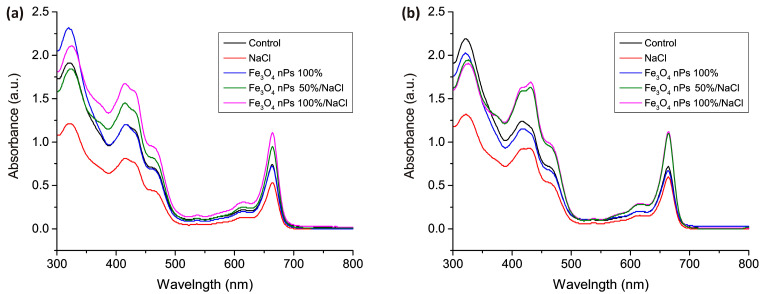
Absorbance measurements for barley samples grown for three weeks (**a**) and four weeks (**b**).

Figure 4 shows the action spectra of the above samples collected after three and four weeks of growth. Table 2 contains numerical data showing the content of chlorophyll *a*, *b*, total chlorophyll, and carotenoids in the samples.

In Figure 4a, it is evident that the action spectrum of the control sample and the sample with added nanoparticles are nearly identical, indicating that the inclusion of nanoparticles does not exert a discernible influence on chlorophyll content, either positively or negatively. Conversely, the action spectrum for NaCl-treated samples is notably lower than the control, signifying a reduction in chlorophyll *a* and chlorophyll *b* concentrations, indicative of impaired plant vital functions under salt stress. Noteworthy observations arise from samples concurrently irrigated with water containing both NaCl and nanoparticles. In these samples, the peaks in the action spectrum exhibit significantly higher values than those observed in samples treated exclusively with NaCl or nanoparticles alone, suggesting a substantial increase in chlorophyll content. Referring to the data in Table 2 and comparing NaCl and nPs 100%/NaCl samples, the increase in chlorophyll a is 107%. Similarly, when comparing the control and nPs 100%/NaCl samples, the increase is approximately 50%. It is evident that the addition of Fe_3_O_4_ nanoparticles positively impacts barley’s ability to withstand salt stress.

This effect may be attributed to magnetite’s capacity to sequester excess Na ions, reducing their concentration in the root zone and preventing their undue penetration into plants. Moreover, nanoparticles can potentially facilitate the transport of essential nutrients and trace elements, enhancing nutrient uptake by plants and compensating for reduced nutrient absorption caused by salt stress. However, the phenomenon where nanoparticles in combination with NaCl yield better results than the control sample, while the addition of nanoparticles alone does not exhibit a similar effect, lacks a clear explanation and necessitates further research. The result of the action spectrum for samples that underwent the influence of the aforementioned factors for an additional week are presented in Figure 4b. Overall, the general trend observed in Figure 4a remains consistent, but it is evident that the disparity in peak heights for the control, NaCl, and nPs samples has diminished. Furthermore, the peak difference between samples containing a 100% concentration of nPs and NaCl and those with a halved concentration of nPs has also vanished, as both graphs are nearly identical. This suggests the presence of an optimal concentration of nanoparticles that, in the long term, may sustain the positive effect of developing salt stress tolerance in plants while simultaneously reducing the nanoparticle concentration. This reduction has the potential to mitigate the future genotoxic impact of nanoparticles and conserve resources expended on their synthesis.

**Figure 5:**
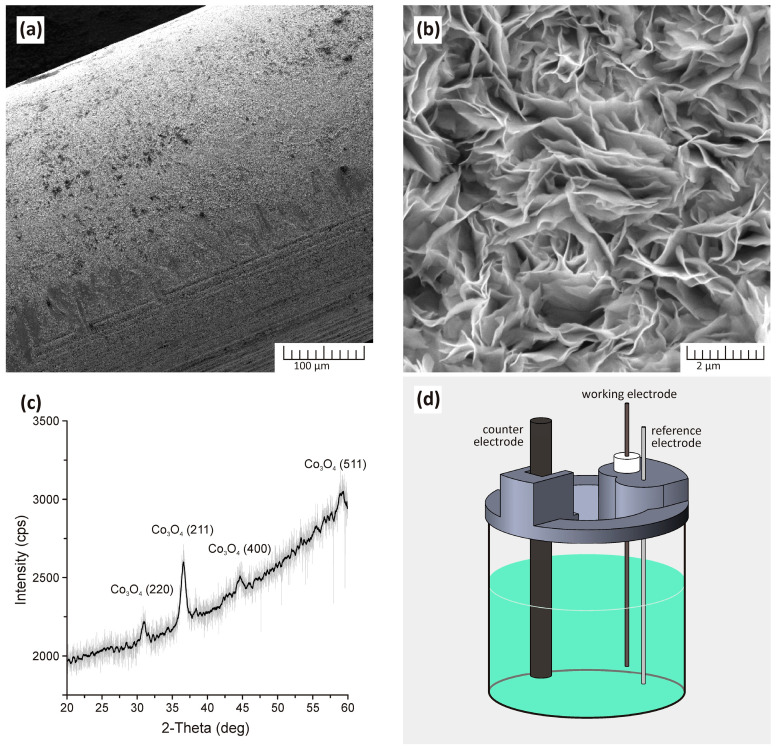
(**a**,**b**) SEM pictures of the resulting nanostructured Co_3_O_4_ coating, where (**a**) is a general view of the iron wire coated with a nanostructured layer and (**b**) is a view of petal-shaped Co_3_O_4_ nanostructures at high magnification. Herein addition, (**c**) displays the XRD spectrum of the crystal structure of the resulting nanostructured coating and (**d**) shows a schematic representation of a three-electrode electrochemical cell, where the above-mentioned iron wire coated with Co_3_O_4_ nanostructures serves as the working electrode.

Figure 5a,b display SEM images of resulting Co_3_O_4_ nanostructures obtained on Fe wire, where Figure 5a is a general view of an electrode indicating a homogeneous covering with nanostructures and Figure 5b displays a detailed view at high magnification of Co_3_O_4_ nanostructures.

This fact indicates that replacing the chlorine-containing cobalt salt precursor with a nitrate-containing one, while keeping other growth parameters unchanged, led to a total change in the morphology of the resulting nanostructures. If in the previous case the coating was a honeycomb network formed from nanofibers, then in this case a dense and uniform coating consisting of 2D petal-shaped nanostructures can be observed on the surface.

X-ray Diffraction (XRD) analysis, as illustrated in Figure 5c, reveals distinctive peaks characteristic of Co_3_O_4_ in the presented nanostructures. Notably, no additional crystalline phases were detected. The pronounced amorphous background is attributed to the predominant composition of the nanostructured coating, primarily comprising thin, vertically oriented petals, with their thinnest section positioned parallel to the surface. In Figure 5d, a generalized schematic illustrates a custom-designed electrochemical cell, featuring a nanostructured wire as the working electrode. The setup includes a glass beaker positioned in a water bath for temperature control. A specially designed lid facilitates secure electrode fixation at a specific height, ensuring consistent electrode length (and, consequently, constant working surface area) across measurements, even after replacing all three electrodes. The electrode is fixed in a sealed holder so that 1 cm of wire is in contact with the solution. The lid also incorporates a sizable central opening, allowing the introduction of the analyzed liquid via a micropipette during measurements and accommodating additional sensors (such as a thermometer or pH meter). To conduct measurements, this cell is linked to the Zahner Zennium electrochemical station.

**Figure 6:**
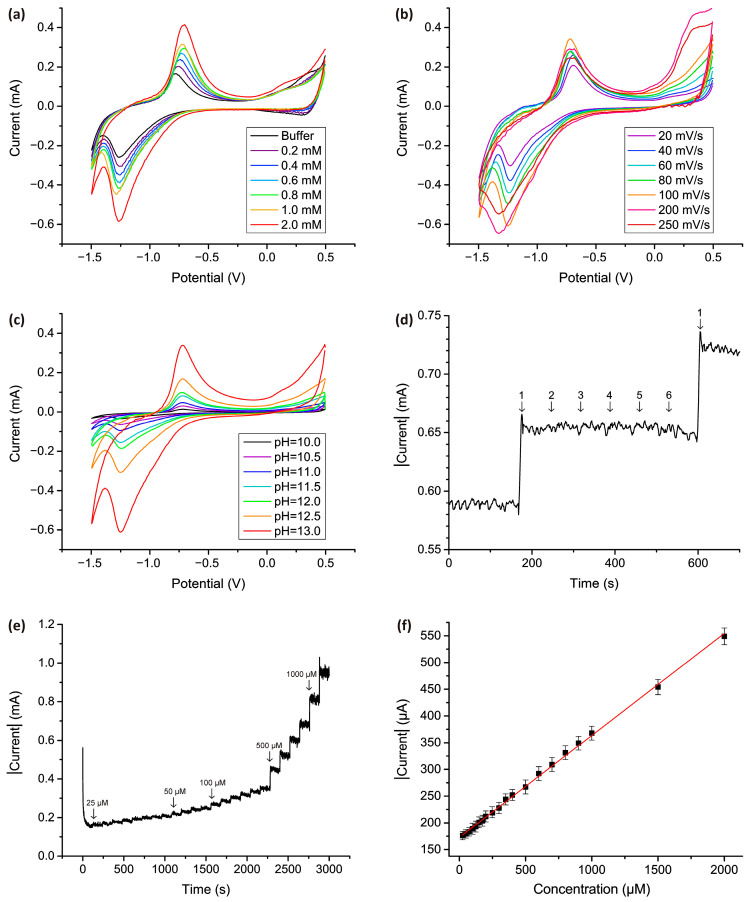
(**a**) CV graph of nanostructured Co_3_O_4_ electrode obtained in a 0.1 M supporting electrolyte and in solutions containing supporting electrolyte and a number of H_2_O_2_ concentrations from 0.2 mM to 2 mM. (**b**) Comparison of CV graphs obtained at different scan speeds. Scanning was performed in a 0.1 M NaOH solution containing 5 mM H_2_O_2_. (**c**) CV measurements performed at different pH values of supporting electrolyte containing 5 mM H_2_O_2_. Scanning was carried out in different concentrations of NaOH solution containing 5 mM H_2_O_2_ at a scanning rate of 100 mV·s^−1^. (**d**) Interference study with the addition of H_2_O_2_ (**1**) and potential interferents NaCl (**2**), KNO_3_ (**3**), glucose (**4**), citric acid (**5**), and ascorbic acid (**6**). (**e**) Chronoamperograms obtained in a 0.1 M NaOH supporting electrolyte for Co_3_O_4_ nanostructured electrode for −1.2 V peak potential obtained by adding H_2_O_2_ in the concentration range from 25 μM to 5 mM. (**f**) Calibration graph for concentration-current dependence.

Figure 6 displays the critical electrochemical measurements, carried out in a supporting electrolyte without the presence of the plant analyte, necessary to determine the characteristics of the sensor and the optimal parameters for its operation.

In the presence of 0.1 M NaOH, the Co_3_O_4_ electrode exhibits two distinct peaks: an anodic peak at approximately −0.7 V and a cathodic peak at approximately −1.23 V. This pair of redox peaks corresponds to a reversible transition between Co_3_O_4_ and CoOOH (as indicated by Equation (11)). As depicted in Figure 6a, the addition of varying concentrations of H_2_O_2_ to the supporting electrolyte induces a pronounced electrochemical response, signifying the occurrence of catalytic processes on the electrode influenced by peroxide. Furthermore, Figure 6a illustrates a direct correlation between the peak height and the added concentration of H_2_O_2_. This reversible electrocatalytic process can be represented by Equation (13):

6CoOOH + H_2_O_2_ → 2Co_3_O_4_ + O_2_↑ + 4H_2_O (13)

Additionally, Figure 6b depicts the impact of scanning speed on the electrochemical response. It is evident that the height of the oxidation peak remains relatively constant with increasing speed, while the height of the reduction peak significantly increases. However, this trend is observable within the range of 20 to 100 mV·s^−1^. Beyond this range, specifically at speeds of 200 mV·s^−1^ and higher, a substantial alteration in peak width and a shift in its maximum are observed, diverging from the anticipated proportional increase in amplitude with scanning speed. Notably, increasing the speed to 250 mV·s^−1^ even results in a decrease in the peak amplitude below the value observed at a speed of 100 mV·s^−1^. Given that a scan rate of 100 mV·s^−1^ offers the maximum electrochemical response without peak shift, this rate was deemed optimal and employed in all subsequent experiments.

The necessity of a supporting electrolyte with a high pH for an effective electrocatalytic process has been previously established in our earlier publication [82]. This phenomenon is elucidated by the presence of hydroxide ions generated through oxyhydroxide formation, which is essential for facilitating the diffusion process within the nanostructured layer. The heightened conductivity, surpassing that of hydroxide, contributes to an improved charge transfer to the wire substrate. Consequently, the application of a negative potential activates the Co_3_O_4_ electrode in an alkaline solution, enabling the successful detection of H_2_O_2_.

In our previous study [77,78], we demonstrated that a pH level of at least 13 was imperative for fiber-like Co_3_O_4_ nanostructures. Even at a pH = 12.5, the reduction peak was not distinctly pronounced. In the case of the petal-shaped nanostructures described in this publication (Figure 6c), measurements reveal that, in contrast to fiber-shaped nanostructures, peaks become evident at a lower pH = 10.5. This suggests a lower sensitivity of this morphology to pH and a reduced dependence of effective electrocatalytic processes on the level of pH. This observation potentially broadens the scope of analytes measurable, allowing for the detection of certain substances where very high pH levels may be undesirable. However, akin to the previous sample, the peak reaches its maximum height at pH = 13, aligning with the 0.1 M NaOH solution employed in these experiments. This choice of supporting electrolyte is thus explained by the consistent performance of the electrocatalytic processes at this pH level.

As previously stated, plant samples comprise a diverse array of components, including solid tissues and a number of organic acids, sugars, and other chemical compounds. Hence, in designing an electrochemical sensor for plant-based substances, it is essential to mitigate the potential for false elevation in the electrochemical signal resulting from potential interferents. To achieve this, interference testing was conducted by introducing substances such as NaCl, KNO_3_, glucose, citric acid, and ascorbic acid, along with H_2_O_2_. As can be seen from Figure 6d, none of the interferons caused a significant electrochemical response, which indicates the high sensitivity of this sensor for the determination of H_2_O_2_ in complex plant analytes.

Figure 6e illustrates the chronoamperogram obtained upon adding H_2_O_2_ to the supporting electrolyte, ranging from 25 µM to 5 mM. It can be seen that to the addition of both small doses (25 µM) of H_2_O_2_ and to the addition of significant doses (500 µM–1mM), an unambiguous and obvious electrochemical response is observed, forming a characteristic step, the height of which depends on the amount of added H_2_O_2_. A calibration curve (Figure 6f) was constructed based on the obtained data, revealing a linear dependence across the entire concentration range. The calculated sensitivity of this sensor is 201 µA·mM^−1^, with a limit of detection (LOD) of 5.2 µM, assuming a signal-to-noise ratio of 3.

Comparing this value with the sensitivity obtained for the fiber-like morphology of Co_3_O_4_, where the sensitivity of the Co_3_O_4_ electrode was 505.11 µA·mM^−1^ and the calculated LOD was 1.05 µM, it is evident that the sensitivity of the petal-shaped morphology is lower in tests conducted in a supporting electrolyte. The decrease in sensitivity may be due to the fact that when the electrode is immersed in a solution, rather thin nanopetals can stick together, forming denser formations, which reduces the working surface area and makes it difficult for liquid to penetrate between the petals. In fiber-like structures, the agglomeration effect was not observed, since nanostructures initially have a larger diameter and a more pronounced shape.

Table 3 presents a comparative analysis of this sensor alongside others documented in the literature that function on a similar principle. The sensor demonstrates a limit of detection (LOD) and sensitivity comparable to some sources, with publications indicating both higher and lower values. It is important to note that for a specific analyte, such sensitivity and LOD values are more than adequate, given that the detectable range of H_2_O_2_ concentrations in plants typically exceeds 5 µM. At this stage, the primary consideration lies in the stable operation of this electrode in plant analytes with complex chemical compositions, enabling its utilization in real sample analysis. If required, future enhancements could focus on augmenting sensitivity by increasing the working surface area of the electrode through the replacement of wire bases with metal plates and refining the geometry of the electrochemical cell.

**Figure 7:**
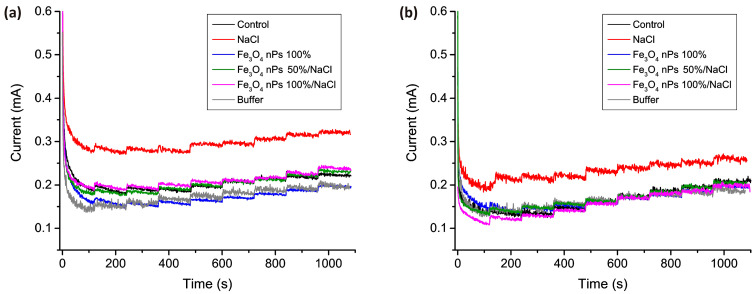
Chronoamperograms of barley extract samples obtained by a stepwise addition of H_2_O_2_ to the test solution in the concentration range from 25 µM to 200 µM with 25 µM increments. Here are two sets of samples: (**a**) samples with a total growth time of 3 weeks, and (**b**) samples with a total growth time of 4 weeks.

Figure 7 presents chronoamperograms obtained from real barley juice samples subjected to salt stress for three and four weeks of growth. The corresponding numerical values of detected H_2_O_2_ are compiled in Table 4, with the “Found” column indicating concentration values directly derived from the graph. The “Excess” column reflects the concentration of H_2_O_2_ formed in the plant, calculated as the difference between the concentration determined during the measurement process and the known manually added concentration of H_2_O_2_.

In Figure 7a, the chronoamperogram for the control sample after 3 weeks of growth, unexposed to salt stress and nanoparticles, aligns with the calibration plot in the maintenance electrolyte. This alignment underscores the accurate and reliable functionality of the sensor, demonstrating that the intricate plant matrix does not interfere with the measurement process, highlighting the sensor’s high selectivity. For the sample exposed to NaCl, a notable excess concentration of H_2_O_2_ (averaging 218 µM) is observed, indicative of significant oxidative stress. Furthermore, the results illustrate that the introduction of Fe_3_O_4_ nanoparticles to a water for irrigation containing NaCl contributes to the development of tolerance to salt stress in barley samples. Both the addition of 100% nanoparticles and a 50% reduction in nanoparticle concentration result in chronoamperograms where additionally released H_2_O_2_ is not observed, aligning with the control sample.

Figure 7b displays chronoamperograms for barley samples subjected to stress for one additional week, extending the total growth duration to 4 weeks. As evident from Figure 7b, only the chronoamperogram obtained for samples treated with pure Fe_3_O_4_ nanoparticles aligns with the calibration graph, substantiating their positive impact on plant viability and resilience to various environmental factors. Contrarily, in all other samples, additional H_2_O_2_ was released over time. Despite the prolonged growth period, the control group exhibits a noteworthy amount of released H_2_O_2_, likely influenced by other stress factors and the natural aging of the first leaf. Notably, in the sample treated solely with NaCl, the released peroxide doubled over the course of a week, averaging 539 µM. The addition of nanoparticles to irrigation water containing NaCl, while not completely eliminating the released H_2_O_2_, reduces its amount to that observed in the control sample. This reduction, akin to the findings in the 3-week samples, signifies the evident development of tolerance to salt stress. Importantly, this positive effect persists even when the nanoparticles introduced into the irrigation water are halved. If we compare the chronoamperograms for barley samples obtained using the petal-shaped morphology of Co_3_O_4_ with the results obtained in a previous publication for the fiber-like morphology, it is clear that this morphology of nanostructures behaves more stably in plant samples. For all samples, the slope remains unchanged and coincides with the slope for the calibration sample obtained on the supporting electrolyte without the addition of the plant analyte. This fact indicates that in the case of this morphology, plant components do not have a negative impact on the operation process and measurement accuracy of this sensor, therefore the petal-shaped morphology of Co_3_O_4_ is more suitable for the analysis of plant samples of complex chemical composition, even despite the sensitivity being lower than was observed with fibrous morphology.

## 4. Conclusions

In summary, this study successfully developed an electrochemical sensor utilizing petal-shaped nanostructures of Co_3_O_4_. The transformation from a fiber-like to a petal-like morphology was achieved by substituting a chlorine-containing precursor with a nitrate-containing precursor. While initial electrochemical measurements revealed lower sensitivity in detecting H_2_O_2_ within a supporting electrolyte, the petal-like morphology demonstrated enhanced stability when applied to real samples. Notably, this morphology mitigated the negative impact of plant analyte matrices, ensuring more consistent and reliable results.

Utilizing this sensor, the study delved into the impact of salt stress on barley seedlings and explored the potential ameliorative effects of Fe_3_O_4_ nanoparticles. The findings indicated that salt stress induced a substantial release of H_2_O_2_ in plants (up to 500 µM), indicative of oxidative stress. However, the introduction of Fe_3_O_4_ nanoparticles into the irrigation water containing NaCl resulted in a reduction of released H_2_O_2_ to levels comparable to the unstressed control sample. This suggests a noteworthy development of salt stress tolerance and the alleviation of oxidative stress in barley facilitated by the nanoparticles.

The positive influence of nanoparticles on oxidative stress reduction, as detected by the electrochemical sensor, was further corroborated by optical absorption measurements. Specifically, samples subjected to NaCl solution exhibited a significant decline in chlorophyll content compared to the control samples. In contrast, samples treated with both NaCl and Fe_3_O_4_ nanoparticles displayed a substantial increase in chlorophyll content, surpassing both the control sample and the NaCl-treated sample without nanoparticles. This observed increase exceeded 50% relative to the control sample and over 100% relative to the NaCl-treated sample without nanoparticles, underscoring the beneficial impact of Fe_3_O_4_ nanoparticles in mitigating the adverse effects of salt stress on barley seedlings. These multifaceted findings collectively underscore the potential of the developed nanopetal Co_3_O_4_ electrochemical sensor and highlight the promising applications of Fe_3_O_4_ nanoparticles in ameliorating oxidative stress in plants exposed to salt stress.

## Figures and Tables

**Table 1:** Morphological parameters of barley samples.

Sample	First Leaf Length (cm), Average and Maximal		Total Length of Green Part (cm), Average and Maximal		Fresh Weight of 10 Plants (g)	Dry Weight of 10 Plants (g)
**Control**	10.5	12.0	21.0	22.0	1.82	0.12
**NaCl**	9.0	10.5	22.0	26.0	1.77	0.14
**nPs 100%**	9.5	12.0	22.0	24.0	1.81	0.15
**nPs 50%/NaCl**	10.0	11.5	24.0	27.0	2.43	0.16
**nPs 100%/NaCl**	11.0	12.0	26.0	29.0	2.37	0.16

**Table 2:** Chlorophyll concentration in barley samples grown under the influence of salt stress and Fe_3_O_4_ nanoparticles.

3 Weeks	A (λ = 645 nm)	A (λ = 663 nm)	A (λ = 480 nm)	Chl(α), mg/g FW	Chl(β), mg/g FW	Chl(α + β), mg/g FW	Carot., mg/g FW
**Control**	0.34	0.74	0.48	0.3393	0.1729	0.5121	0.0139
**NaCl**	0.22	0.53	0.29	0.2456	0.1023	0.3478	0.0084
**nPs 100%**	0.33	0.72	0.45	0.3303	0.1675	0.4976	0.0129
**nPs 50%/NaCl**	0.39	0.94	0.55	0.4356	0.1813	0.6167	0.0163
**nPs 100%/NaCl**	0.47	1.1	0.64	0.5082	0.2246	0.7326	0.0186
**4 weeks**	**A (λ = 645 nm)**	**A (λ = 663 nm)**	**A (λ -= 480 nm)**	**Chl(α),** **mg/g FW**	**Chl(β),** **mg/g FW**	**Chl(α + β), mg/g FW**	**Carot., mg/g FW**
**Control**	0.32	0.71	0.48	0.3262	0.1602	0.4863	0.0143
**NaCl**	0.25	0.59	0.36	0.2728	0.1186	0.3913	0.0107
**nPs 100%**	0.31	0.67	0.47	0.3070	0.1585	0.4654	0.0139
**nPs 50%/NaCl**	0.43	1.09	0.67	0.5075	0.1898	0.6971	0.0208
**nPs 100%/NaCl**	0.44	1.11	0.69	0.5165	0.1952	0.7116	0.0214

**Table 3:** Analytical performance of the obtained nanopetal-based Co_3_O_4_ electrochemical sensor compared to other reported non-enzymatic H_2_O_2_ sensors.

Electrode	Sensitivity	Linear Range	LOD	Reference
**Co_3_O_4_/TiO_2_ NTs**	39.53 μA·mM^−1^·cm^−2^	1.27–26.80 mM	6.71 μM	[83]
**[Co(pbda)(4,4-bpy)(2H_2_O)]n/GCE**	83.10 μA·mM^−1^·cm^−2^	50–9000 μM	3.76 μM	[84]
**Co_3_O_4_ /MWCNTs/CPE**	729.7 μA·mM^−1^	20–430 μM	2.46 μM	[85]
**Ni(OH)_2_ nPs**	1660 μA·mM^−1^·cm^−2^	30–320 μM	26.4 μM	[86]
**CuO/CoO**	6349 μA·mM^−1^	2–4000 μM	1.4 μM	[87]
**CoO-CoS/NF**	590 μA·mM^−1^	2–954 μM	0.890 μM	[88]
**MnOx/CNW**	698.6 μA·mM^−1^· cm^−2^	40–10.230 μM	0.55 μM	[89]
**Co_3_O_4_ nPTLS**	201 µA·mM^−1^	25–5000 μM	5.2 µM	This work

**Table 4:** H_2_O_2_ determination in barley samples grown under the influence of salt stress and Fe_3_O_4_ nanoparticles.

**3 Weeks**								
**Buffer**			**Control**			**NaCl**		
Added (µM)	Found (µM)	Excess (µM)	Added (µM)	Found (µM)	Excess (µM)	Added (µM)	Found (µM)	Excess (µM)
25	25	0	25	30	5	25	221	196
50	50	0	50	25	−25	50	237	187
75	75	0	75	60	−15	75	250	175
100	100	0	100	102	2	100	320	220
125	125	0	125	129	4	125	348	223
150	150	0	150	160	10	150	379	229
175	175	0	175	200	25	175	424	249
200	200	0	200	230	30	200	454	254
**nPs 100%**			**nPs 50%/NaCl**			**nPs 100%/NaCl**		
Added (µM)	Found (µM)	Excess (µM)	Added (µM)	Found (µM)	Excess (µM)	Added (µM)	Found (µM)	Excess (µM)
25	28	3	25	30	5	25	0	−25
50	55	5	50	65	15	50	30	−20
75	70	−5	75	80	5	75	60	−15
100	107	7	100	105	5	100	95	−5
125	125	0	125	129	4	125	133	8
150	155	5	150	160	10	150	155	5
175	185	10	175	200	25	175	193	18
200	220	20	200	230	30	200	210	10
**4 weeks**								
**Buffer**			**Control**			**NaCl**		
Added (µM)	Found (µM)	Excess (µM)	Added (µM)	Found (µM)	Excess (µM)	Added (µM)	Found (µM)	Excess (µM)
25	25	0	25	82	57	25	561	536
50	50	0	50	103	53	50	578	528
75	75	0	75	93	28	75	590	515
100	100	0	100	151	51	100	630	530
125	125	0	125	169	44	125	643	518
150	150	0	150	213	63	150	692	542
175	175	0	175	241	66	175	756	581
200	200	0	200	261	61	200	777	577
**nPs 100%**			**nPs 50%/NaCl**			**nPs 100%/NaCl**		
Added (µM)	Found (µM)	Excess (µM)	Added (µM)	Found (µM)	Excess (µM)	Added (µM)	Found (µM)	Excess (µM)
25	15	−10	25	70	45	25	113	88
50	44	−6	50	96	46	50	115	65
75	50	−25	75	103	28	75	139	64
100	76	−24	100	154	54	100	184	84
125	98	−27	125	168	43	125	170	45
150	135	−15	150	213	63	150	213	63
175	170	−5	175	225	50	175	291	116
200	190	−10	200	298	98	200	347	147

## Data Availability

Data is contained within the article.

## Appendix A

**Table A1:** Full data of EDS results for mineral content in barley samples.

Element	Control (Weight %)	NaCl (Weight %)	nPs 100% (Weight %)	nPs 50%/NaCl (Weight %)	nPs 100%/NaCl (Weight %)
C	53.11	50.40	50.23	50.59	49.59
O	37.19	32.22	40.41	39.19	37.19
Na	0.31	3.43	0.20	0.99	2.25
Mg	0.61	0.25	0.77	0.67	0.65
Si	0.09	0.04	0.04	0.37	0.23
P	1.26	0.66	1.30	0.79	0.85
S	1.40	0.54	1.07	0.70	0.46
Cl	0.44	7.77	0.54	3.71	4.86
K	3.97	3.96	3.75	1.22	2.58
Ca	1.46	0.60	1.54	1.55	1.17
Fe	0.04	0.02	0.06	0.10	0.06
Cu	0.12	0.11	0.09	0.12	0.11
Total	100.00	100.00	100.00	100.00	100.00
